# Prognostic value of metabolic tumor volume of pretreatment ^18^F-FAMT PET/CT in non-small cell lung Cancer

**DOI:** 10.1186/s12880-018-0292-2

**Published:** 2018-11-26

**Authors:** Soma Kumasaka, Takahito Nakajima, Yukiko Arisaka, Azusa Tokue, Arifudin Achmad, Yasuhiro Fukushima, Kimihiro Shimizu, Kyoichi Kaira, Tetsuya Higuchi, Yoshito Tsushima

**Affiliations:** 1 Department of Diagnostic Radiology and Nuclear Medicine, Gunma University Graduate School of Medicine, Showa-machi 3-39-22, Maebashi, Gunma 371-8511 Japan; 2 Department of Nuclear Medicine and Molecular Imaging Faculty of Medicine, Universitas Padjadjaran Jalan Professor Eyckman No.38, Bandung, Jawa Barat 40161 Indonesia; 3 Current affiliation is Division of Clinical Radiology Service, Kyoto University Hospital, 54 Kawaharacho, Shogoin, Sakyo-ku, Kyoto, 606-8507 Japan; 4 Department of Thoracic and Visceral Organ Surgery, Gunma University Graduate School of Medicine, Showa-machi 3-39-22, Maebashi, Gunma 371-8511 Japan; 5 Department of Oncology Clinical Development, Gunma University Graduate School of Medicine, Showa-machi 3-39-22, Maebashi, Gunma 371-8511 Japan

**Keywords:** PET, Metabolic tumor volume, Lung cancer, Prognosis, ^18^F-FAMT PET/CT

## Abstract

**Background:**

This study aimed to determine the prognostic value of positron emission tomography (PET) metabolic parameters—namely metabolic tumor volume (MTV), total lesion glycolysis (TLG), and total lesion retention (TLR)—on fluorine-18 (^18^F) fluorodeoxyglucose (FDG) and L- [3-^18^F]-α-methyltyrosine (^18^F-FAMT) PET/CT in patients with non-small-cell lung cancer (NSCLC).

**Methods:**

The study group comprised 112 NSCLC patients who underwent ^18^F-FDG and ^18^F-FAMT PET/CT prior to any therapy. The MTV, TLG, TLR, and maximum standardized uptake value (SUV_max_) of the primary tumors were determined. Automatic MTV measurement was performed using PET volume computer assisted reading software. (GE Healthcare). Cox proportional hazards models were built to assess the prognostic value of MTV, TLG (for ^18^F-FDG), TLR (for ^18^F-FAMT), SUV_max_, T stage, N stage, M stage, clinical stage, age, sex, tumor histological subtype, and treatment method (surgery or other therapy) on overall survival (OS).

**Results:**

Higher TNM, higher clinical stage, inoperable status, and higher values for all PET parameters (both ^18^F-FAMT and ^18^F-FDG PET) were significantly associated (*P* < 0.05) with shorter OS. Multivariate analysis revealed that a higher MTV of ^18^F-FAMT (hazard ratio [HR]: 2.88, CI: 1.63–5.09, *P* < 0.01) and advanced clinical stage (HR: 5.36, CI: 1.88–15.34, *P* < 0.01) were significant predictors of shorter OS.

**Conclusions:**

MTV of ^18^F-FAMT is of prognostic value for OS in NSCLC cases and can help guide decision-making during patient management.

## Background

Lung cancer is the leading cause of cancer-related death worldwide for both men and women. Non-small-cell lung cancer (NSCLC) accounts for 80% of all lung cancers [1]. Despite progress in treatment strategies, overall survival (OS) in NSCLC remains unacceptably short—even for early-stage disease—and progressively worsens with increasing TNM stage [2, 3]. Currently, TNM stage is one of the most important prognostic factors for NSCLC and serves a valuable guide when choosing a treatment strategy [3, 4]. However, TNM staging alone does not always provide satisfactory results because each stage consists of a heterogeneous population with a different risk of relapse. Therefore, improved methods are needed to accurately predict prognosis and guide treatment strategy.

Fluorine-18 (^18^F) fluorodeoxyglucose (FDG) positron emission tomography (PET) is widely used for initial staging, restaging at recurrence, estimating radiotherapeutic or chemotherapeutic responses, and delineating radiotherapeutic targets [5–10]. The standardized uptake value (SUV) in the NSCLC primary lesion at the time of diagnosis is known to be an important prognostic factor [11].

Maximum SUV (SUV_max_) is a long-established value in clinical practice for quantifying a lesion’s metabolism. However, as it is based on a single voxel value, SUV_max_ may not represent total tumor metabolism. By contrast, PET metabolic parameters, such as metabolic tumor volume (MTV) and total lesion glycolysis (TLG), are volumetric indices, and are thus more reliable reflections of tumor burden and aggressiveness [12]. Furthermore, these metabolic parameters are potentially useful prognostic markers for various malignancies examined by ^18^F-FDG PET [13–16].

We have developed l-[3-^18^F]-α–methyltyrosine (^18^F-FAMT), an amino acid PET tracer that specifically accumulates in tumor cells via L-type amino acid transporter 1 (LAT1) [17–20]. In the last two decades, ^18^F-FAMT has been investigated in various tumors and shown to offer some additional clinical benefits over ^18^F-FDG [21, 22]. ^18^F-FAMT uptake within the primary tumor, as depicted by SUV_max_, is associated with poor outcomes in NSCLC patients and is a stronger prognostic factor than ^18^F-FDG uptake [23], making it useful for diagnosis, staging [19], and assessment of therapeutic response [24]. Therefore, in this study we postulated that the metabolic tumor burden, as indicated by MTV and total lesion retention (TLR) of ^18^F-FAMT, is useful as an indicator of prognosis. The purpose of this study was to determine the prognostic value of PET metabolic parameters (namely MTV, TLG, and TLR) on ^18^F-FDG and ^18^F-FAMT PET/CT in patients with NSCLC.

## Methods

### Patient selection

The medical records of 112 consecutive NSCLC patients at our institution between April 2007 and August 2013 who underwent both ^18^F-FAMT and ^18^F-FDG PET/CT before receiving any therapy were retrospectively reviewed. Clinical and pathological TNM stages were established using the Union Internationale Centre le Cancer (UICC) classification. All patients agreed to participate in this study and provided written informed consent. The institutional review board approved the study protocol. Thirteen of the 112 patients have been included in previous reports [19, 23, 25]. These previous articles solely evaluated SUV_max_ of ^18^F-FAMT or LAT1 expression of the tumor, whereas this study evaluated PET metabolic parameters (MTV and TLR) and survival prognosis.

### Tracer preparation and PET scan acquisition

^18^F-FAMT was synthesized in our hospital cyclotron facility according to the method developed by Tomiyoshi et al. [17]. The radiochemical yield of ^18^F-FAMT was approximately 20%, with a radiochemical purity of approximately 99%. Molar activity of ^18^F-FAMT exceeded 0.12 GBq /μmol (3.24 Ci /mmol). ^18^F-FDG was also produced in our facility as previously described [19]. Patients fasted for at least six hours prior to ^18^F-FDG PET imaging. Patients were then injected with 5 MBq/kg of ^18^F-FAMT or 5 MBq/kg of ^18^F-FDG and PET acquisition was performed one hour later. One of two PET/CT scanners (Discovery STE 16, GE Healthcare, Milwaukee, USA; Biograph 16 Siemens Medical Solutions, Erlangen, Germany) was randomly selected for PET/CT acquisition. Scan parameters are shown in Table 1.

**Table 1:** Protocol parameters for PET/CTs

parameters	Discovery STE 16	Biograph 16
PET scan parameters		
FOV of PET (mm)	700 × 700	700 × 700
slice thickness of PET (mm)	3.27	2.0
acquisition time (sec/bed)	120	120
bed number (bed/body)	5–6	5–6
matrix of PET (pixel)	128 × 128	128 × 128
energy window range (keV)	425–650	425–650
reconstruction	3D Iterative Reconstruction	OSEM 2D
CT scan parameters		
FOV of CT (mm)	500	500
slice thickness of CT (mm)	3.75	5
matrix of CT (pixel)	512 × 512	512 × 512
X-ray tube voltage (kVp)	120	120
X-ray tube current (mA)	60	400

### PET/CT analysis and tumor volume measurement

Two experienced nuclear medicine physicians (T.H., Y.A.) interpreted all ^18^F-FAMT and ^18^F-FDG PET images. Pre-existing PET data were re-analyzed for MTV, TLG, and TLR. PET VCAR (Volume Computer Assisted Reading) software on an Advantage Workstation (GE Healthcare, Milwaukee, WI) was used to automatically calculate the MTV of each lesion using SUV thresholds of 1.2 for ^18^F-FAMT and 2.5 for ^18^F-FDG. SUV_max_. The average SUV (SUV_mean_) within the generated 3D volume of interest (VOI) was also calculated automatically. TLG or TLR was defined as MTV multiplied by SUV_mean_. For patients with metastases, PET parameters were determined only by their primary tumors.

### Statistical analysis

OS was defined as the time from initial PET/CT examination until patient death from any cause. For survivors, survival time was censored at the last date that the patient was known to be alive. Time-to-progression and progression-free survival was not evaluated because the times for subsequent PET imaging varied between patients. Survival analysis was carried out using the Kaplan-Meier method with a log-rank test to assess differences in patient survival between high and low values of the PET parameters. The median value for each PET parameter was employed as a cut-off in the subsequent analysis.

Univariate and multivariate analyses were performed using Cox proportional hazard models to identify the independent prognostic factors for OS. The prognostic factors analyzed included MTV, TLG (for ^18^F-FDG), TLR (for ^18^F-FAMT), SUV_max_, T stage, N stage, M stage, clinical stage, age, sex, tumor histological subtype, and treatment method (surgery or other therapy). In the multivariate analysis, all variables except T stage, N stage, and M stage were included, while the forward stepwise method was applied to assess the potential independent effects of prognostic factors for OS. All statistical analyses were performed using SPSS Statistics Version 21.0 (IBM Corp. Released 2012. Armonk, NY: IBM Corp.). A *P* value of 0.05 was selected as the threshold of statistical significance.

## Results

**Fig. 1:**
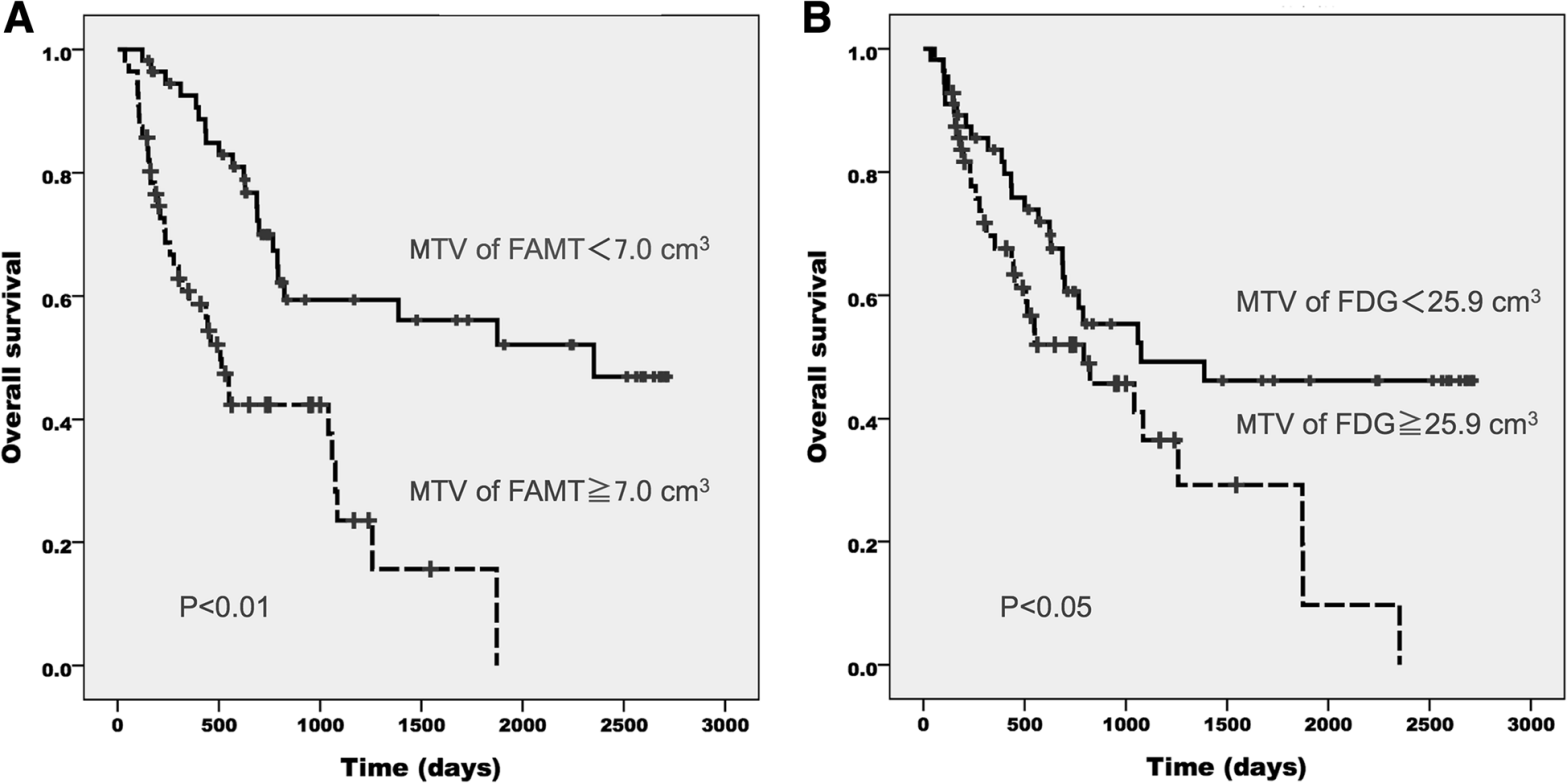
Overall survival of NSCLC patients according to the MTV of ^18^F-FAMT (**a**) and ^18^F-FDG (**b**)

**Fig. 2:**
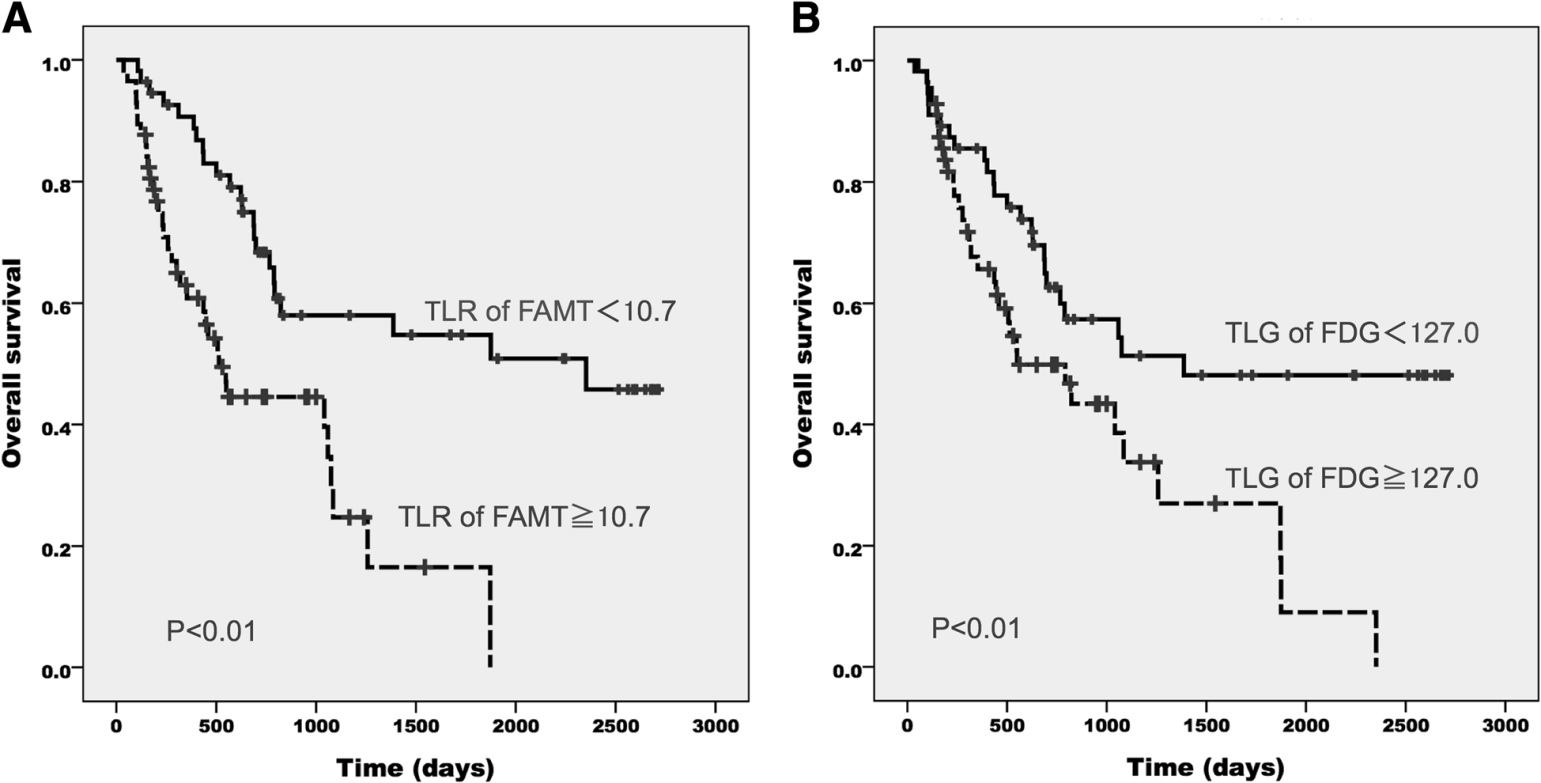
Overall survival of NSCLC patients according to the TLR of ^18^F-FAMT (**a**) and TLG of ^18^F-FDG (**b**)

**Fig. 3:**
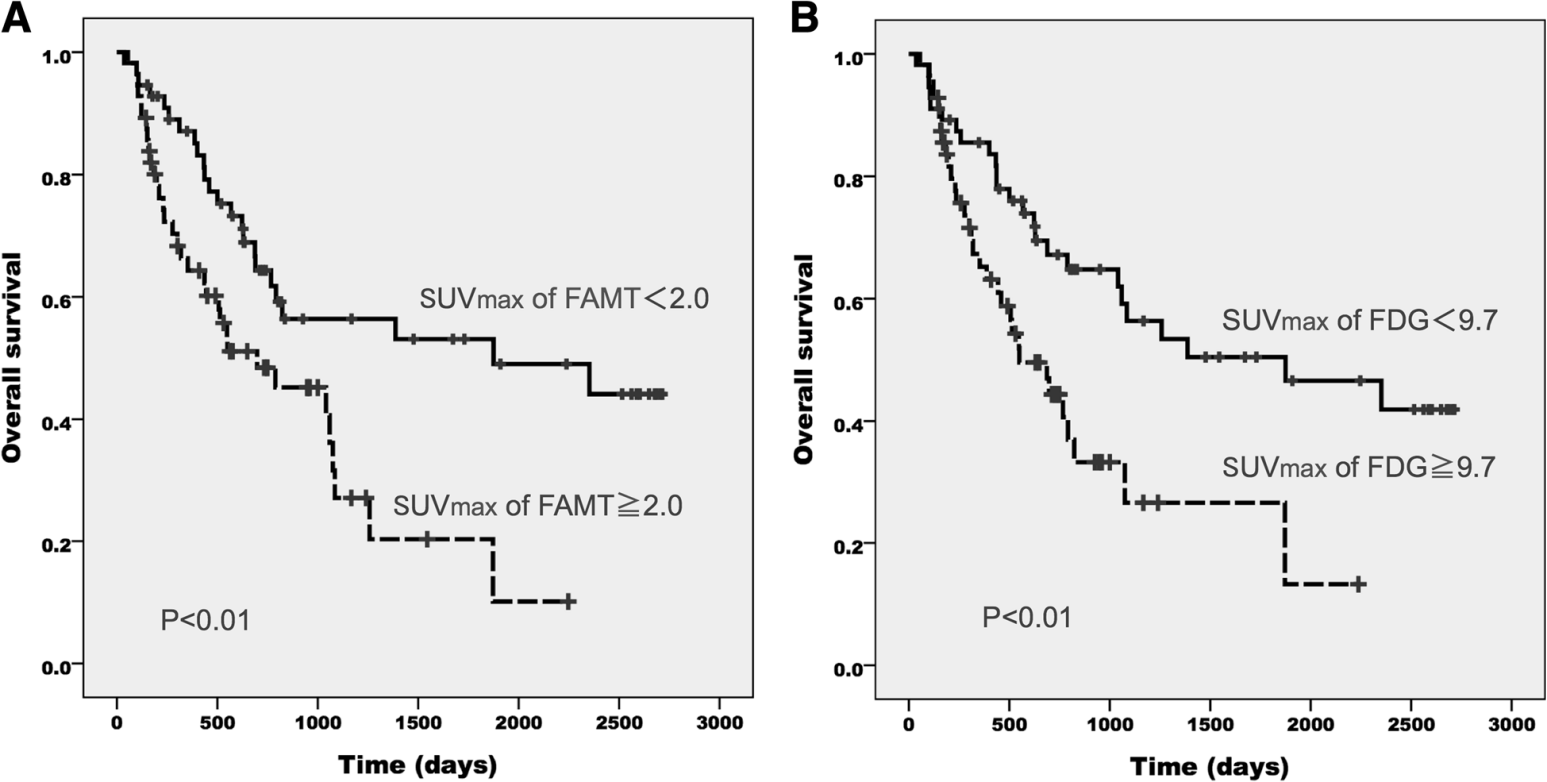
Overall survival of NSCLC patients according to the SUV_max_ of ^18^F-FAMT (**a**) and ^18^F-FDG (**b**)

The study involved 112 patients (84 males, 28 females) with a median age of 69 years (range 32–85 years). A summary of patient and tumor characteristics is presented in Table 2. The median time interval between ^18^F-FDG PET and ^18^F-FAMT PET was 3 days (mean, 5.8; range, 1–32 days). Seventy patients underwent ^18^F-FDG PET prior to ^18^F-FAMT PET (70/112 cases, 62.5%), while 42 patients underwent ^18^F-FAMT PET before ^18^F-FDG PET. The median SUV_max_, MTV, and TLR (or TLG) values were 2.0, 7.0 cm^3^, and 10.7 for ^18^F-FAMT and 9.7, 25.9 cm^3^, and 127.0 for ^18^F-FDG, respectively. The median follow-up duration at the end of the study was 575.5 days. Fifty-five patients (49%) were alive at the end of the follow-up period. All PET parameters of both radiotracers significantly differentiated patient OS based on the respective cut-off values (Figs. 1, 2 and 3). Patients with larger MTV had a significantly shorter median OS than those with smaller MTV on both ^18^F-FAMT (507 days vs. 2352 days) (Fig. 1a) and ^18^F-FDG (792 days vs. 1075 days) (Fig. 1b).

**Table 2:** Summary of Patients Characteristics

Variable	All patients (*n* = 112)
Median age (years)^a^	69 (32–85)
Sex, n (%)	
M	84 (75.0%)
F	28 (25.0%)
Histologic subtype, n (%)	
Adenocarcinoma	72 (64.3%)
Squamous cell carcinoma	28 (25.0%)
Other	12 (10.7%)
TNM stage	
T1/T2/T3/T4	25 / 47 / 10 / 30
N0/N1/N2/N3	29 / 13 / 41 / 29
M0/M1	63 / 49
Stage I / II / III / IV	16 / 1 / 47 / 48
Treatment, n (%)	
Chemotherapy	82 (73.2%)
Surgery	21 (18.8%)
Radiation	1 (0.9%)
Surgery + Chemotherapy	8 (7.1%)

^a^Numbers in parentheses are ranges

In the univariate Cox proportional hazard model analyses, a higher TNM, higher clinical stage, inoperable status, and higher values for all ^18^F-FAMT and ^18^F-FDG PET parameters were significantly associated with shorter OS (*P* < 0.05). Older age, male sex, and adenocarcinoma subtypes were not associated with shorter OS. However, when all factors except T stage, N stage, and M stage were included in the forward stepwise multivariate Cox proportional hazard model, two independent significant prognostic factors of OS remained: MTV of ^18^F-FAMT (hazard ratio [HR]: 2.88, CI: 1.63–5.09, *P* < 0.01) and clinical stage (HR: 5.36, CI: 1.88–15.34, *P* < 0.01). The results of the univariate and multivariate analyses of factors affecting OS are summarized in Table 3.

**Table 3:** Cox proportional Hazard model analysis of potential prognostic factors influencing Os

parameters	Univariate Analysis		Multivariate Analysis	
	Hazard Ratio (95% CI)	*p* value	Hazard Ratio (95% CI)	*p* value
Patient				
age (69 vs < 69)	1.17 (0.69, 1.96)	0.57		
sex (Male vs Female)	1.44 (0.76, 2.73)	0.26		
Histologic subtype				
adenocarcinoma vs others	0.80 (0.47, 1.37)	0.42		
TNM stage				
T stage (T3/4 vs T1/2)	2.57 (1.49, 4.44)	< 0.01		
N stage (N2/3 vs N0/1)	1.84 (1.03, 3.25)	< 0.05		
M stage (M1 vs M0)	2.20 (1.28, 3.77)	< 0.01		
Clinical stage				
III/IV vs I/II	5.92 (2.08, 16.80)	< 0.01	5.36 (1.88, 15.34)	< 0.01
Treatment				
inoperable vs operable	5.37 (2.31, 12.45)	< 0.01		
^18^F-FDG PET parameters				
SUV_max_ (9.7 vs < 9.7)	2.24 (1.29, 3.88)	< 0.01		
MTV (cm^3^) (25.9 vs < 25.9)	1.81 (1.06, 3.08)	< 0.05		
TLG (127.0 vs < 127.0)	2.03 (1.19, 3.48)	< 0.05		
^18^F-FAMT PET parameters				
SUV_max_ (2.0 vs < 2.0)	2.17 (1.26, 3.74)	< 0.01		
MTV (cm^3^) (7.0 vs < 7.0)	3.14 (1.79, 5.53)	< 0.01	2.88 (1.63, 5.09)	< 0.01
TLR (10.7 vs < 10.7)	2.78 (1.59, 4.87)	< 0.01		

## Discussion

In the present study, MTV of ^18^F-FAMT was found to be highly prognostic of OS in NSCLC cases, regardless of tumor subtype and stage. The clinical stage remained as an independent prognostic factor of OS along with MTV. Previous meta-analysis has shown that ^18^F-FDG uptake, as represented by SUV_max_, in the primary tumors of NSCLC patients, is an independent prognostic factor for survival [11]. However, in this study, SUVmax of ^18^F-FAMT and ^18^F-FDG was not an independent prognostic factor of OS. One possibility for this result is that when a tumor reaches an advanced stage, SUVmax, which is a single voxel representation, is no longer prognostic.

Several studies have found that the volumetric parameter is potentially a better predictor of outcome than SUV_max_ [26–28]. We confirmed that MTV and TLG of ^18^F-FDG failed to serve as independent prognostic factors for NSCLC cases, although recent studies [15, 28–30] and a meta-analysis [12] suggest otherwise. The heterogeneity of the patient populations and different methods used to obtain MTV values might account for this discrepancy. Interestingly, we also found that TLR was not an independent prognostic factor, whereas MTV of ^18^F-FAMT remained significant. This result may relate to the fact that SUV_mean_ of ^18^F-FAMT is typically low and TLR, defined as MTV multiplied by SUV_mean_, might underestimate the tumor volume.

**Fig. 4:**
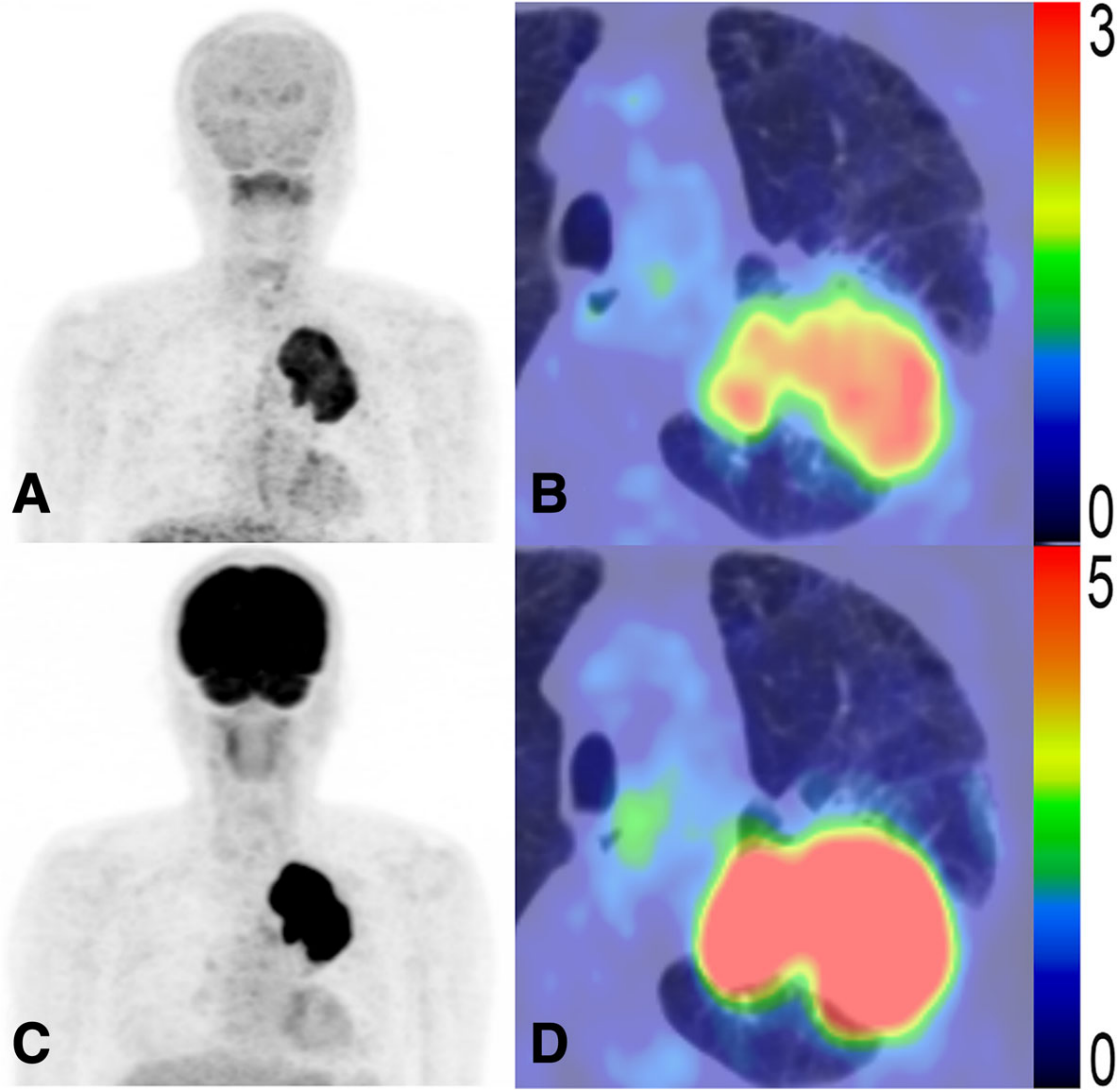
PET images of a 60-year-old male NSCLC patient at stage IIIB (T4N0M0) with high ^18^F-FAMT uptake (SUV_max_ = 3.6, MTV = 166.0 cm^3^, TLR = 315.4) (**a**, **b**) and high ^18^F-FDG uptake (SUV_max_ = 14.8, MTV = 209.0 cm^3^, TLG = 1442.1) (**c**, **d**). This patient was treated with chemotherapy and died 122 days later

**Fig. 5:**
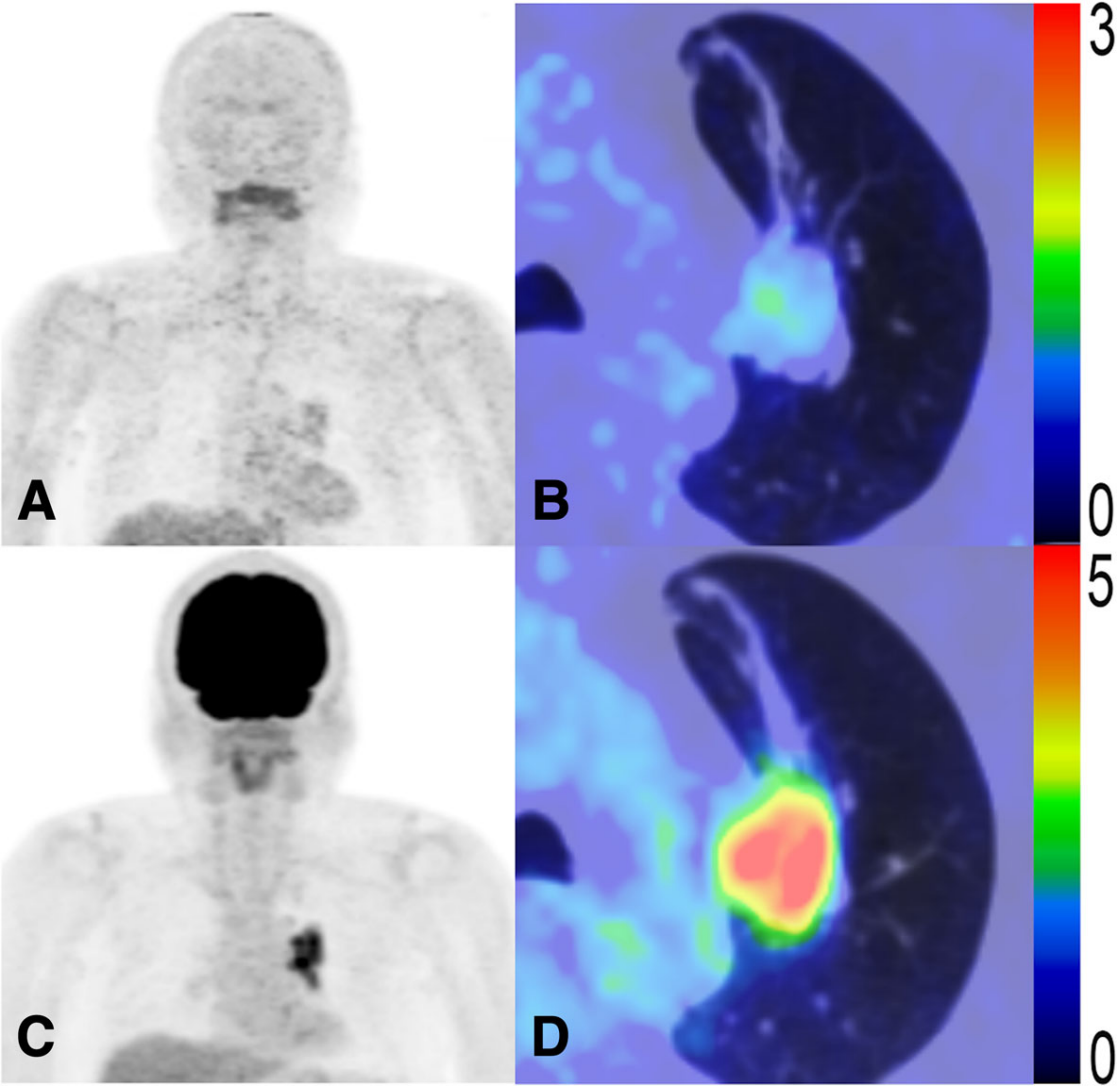
PET images of a 72-year-old male NSCLC patient at stage IIIA (T2N2M0) with low ^18^F-FAMT uptake (SUV_max_ = 1.4, MTV = 0.3 cm^3^, TLR = 0.4) (**a**, **b**) and high ^18^F-FDG uptake (SUV_max_ = 8.8, MTV = 36.0 cm^3^, TLG = 143.7) (**c**, **d**). This patient was treated with surgery combined with chemotherapy and died 1875 days later

This study mainly examined the prognostic potential of MTV and TLR of ^18^F-FAMT, a tumor-specific PET radiotracer. Representative patient images, as shown in Figs. 4 and 5, suggest that ^18^F-FAMT uptake represents malignancy more accurately than ^18^F-FDG uptake, based on patient OS. Our results suggest that MTV of ^18^F-FAMT might have an advantage over MTV of ^18^F-FDG, whereas the independent prognostic value of SUV_max_ for both radiotracers remains questionable. MTV and TLG of ^18^F-FDG have been evaluated in various tumors within the last decade and found to have potential for treatment evaluation or as a prognostic tool [31, 32]. However, ^18^F-FDG has inherent limitations; for instance, physiological uptake and inflammatory uptake may complicate tumor delineation and, in turn, the construction of MTV. The considerable time and effort required to produce MTV—especially if using the manual method—preclude these metabolic parameters from entering daily clinical practice. However, several automated 3D VOI generating software packages have recently been developed to address this challenge [31–33].

The availability of tumor-specific PET radiotracers multiplies the benefits of these metabolic parameters. The present study is the first to evaluate the prognostic value of MTV and TLR on pretreatment ^18^F-FAMT PET/CT in patients with NSCLC. Previous reports have shown the advantage of MTV of ^18^F-FAMT for tumor delineation for accurate volume prediction [21, 34]. Indeed, we found that MTV of ^18^F-FAMT was useful for prognostic purposes.

However, our study had several limitations. The first is the predefined threshold method for delineation of lesion edges; this threshold choice greatly influenced the measurement of MTV, TLG, and TLR. The threshold of SUV 2.5 for ^18^F-FDG was chosen because it is widely used for tumor delineation [12]. For ^18^F-FAMT in NSCLC lesions, this was the first study to comprise tumor volume rather than a single-pixel SUV_max_ value. Thus, we investigated thresholds from SUV 1.2 to 1.8 in a smaller number of patients in advance to determine the optimum threshold for ^18^F-FAMT; SUV 1.2 was found to be optimal for generating a 3D VOI that covered the whole tumor mass in all cases. Second, patients were examined using two different PET/CT scanners. Second, the patients were examined with two different PET/CT scanners. This might have affected the quantitative accuracy of PET data. However, both scanners are routinely cross-calibrated to ensure the comparability of SUV in our hospital. Third, actual tumor uptake of ^18^F-FAMT was relatively low relative to that of ^18^F-FDG [35]. Low uptake may induce false-negative findings if it is used as a single tool for NSCLC staging. However, at our hospital, ^18^F-FAMT PET/CT studies are always performed along with ^18^F-FDG PET/CT. We believe that ^18^F-FAMT PET/CT can provide additional information to what ^18^F-FDG PET/CT provides. Since molecular targeting therapy needs additional information on amino acid metabolism, ^18^F-FAMT PET/CT can provide important information for predicting therapeutic effects. Fourth, only primary tumors were evaluated in patients with metastases. Metabolic information about metastatic tumors may be of additional prognostic value. Thus, MTV, TLG, and TLR may have been underestimated in some cases; however, MTV of ^18^F-FAMT was proven to be a good prognostic indicator. We presumed that metabolic information about metastatic tumors might be insufficient to interfere with the prognostic value of MTV of ^18^F-FAMT. Fifth, this study involved a relatively small number of patients with heterogeneous characteristics at clinical stage I to IV. A study with a larger number of patients is required to further validate our results.

## Conclusion

The MTV of ^18^F-FAMT was found to be an independent risk factor and may be a better predictor of OS than ^18^F-FDG in NSCLC cases. Thus, the MTV of ^18^F-FAMT could be valuable for guiding decision-making during NSCLC patient management.

## Abbreviations

^18^F-FAMT: L- [3-^18^F]-α-methyltyrosine
^18^F-FDG: Fluorine-18-fluorodeoxyglucose
MTV: Metabolic tumor volume
NSCLC: Non-small-cell lung cancer
OS: Overall survival
PET: Positron emission tomography
SUV_max_: Maximum standardized uptake value
TLG: Total lesion glycolysis
TLR: Total lesion retention
VOI: Volume of interest

## References

[1] Navada S, Lai P, Schwartz A, Kalemkerian G (2006). Temporal trends in small cell lung cancer: analysis of the National Surveillance, Epidemiology, and End Results Database. J Clin Oncol..

[2] Woodard GA, Jones KD, Jablons DM (2016). Lung Cancer staging and prognosis. Cancer Treat Res.

[3] Chansky K, Detterbeck FC, Nicholson AG, Rusch VW, Vallières E, Groome P (2017). The IASLC lung Cancer staging project: external validation of the revision of the TNM stage groupings in the eighth edition of the TNM classification of lung Cancer. J Thorac Oncol.

[4] van Rens MT, de la Riviere AB, Elbers HR, van Den Bosch JM (2000). Prognostic assessment of 2,361 patients who underwent pulmonary resection for non-small cell lung cancer, stage I, II, and IIIA. Chest.

[5] Hicks RJ, Kalff V, MacManus MP, Ware RE, Hogg A, McKenzie AF (2001). (18) F-FDG PET provides high-impact and powerful prognostic stratification in staging newly diagnosed non-small cell lung cancer. J Nucl Med.

[6] Hicks RJ, Kalff V, MacManus MP, Ware RE, McKenzie AF, Matthews JP (2001). The utility of (18) F-FDG PET for suspected recurrent non-small cell lung cancer after potentially curative therapy: impact on management and prognostic stratification. J Nucl Med.

[7] Ryu JS, Choi NC, Fischman AJ, Lynch TJ, Mathisen DJ (2002). FDG-PET in staging and restaging non-small cell lung cancer after neoadjuvant chemoradiotherapy: correlation with histopathology. Lung Cancer.

[8] Hoekstra CJ, Stroobants SG, Hoekstra OS, Vansteenkiste J, Biesma B, Schramel FJ (2003). The value of [18F] fluoro-2-deoxy-D-glucose positron emission tomography in the selection of patients with stage IIIA-N2 non-small cell lung cancer for combined modality treatment. Lung Cancer.

[9] Vanuytsel LJ, Vansteenkiste JF, Stroobants SG, De Leyn PR, De Wever W, Verbeken EK (2000). The impact of (18) F-fluoro-2-deoxy-D-glucose positron emission tomography (FDG-PET) lymph node staging on the radiation treatment volumes in patients with non–small cell lung cancer. Radiother Oncol.

[10] Nestle U, Kremp S, Grosu AL (2006). Practical integration of [18F]-FDG-PET and PET-CT in the planning of radiotherapy for non-small cell lung cancer (NSCLC): the technical basis, ICRU-target volumes, problems, perspectives. Radiother Oncol.

[11] Paesmans M, Berghmans T, Dusart M, Garcia C, Hossein-Foucher C, Lafitte JJ (2010). Primary tumor standardized uptake value measured on fluorodeoxyglucose positron emission tomography is of prognostic value for survival in non-small cell lung cancer: update of a systematic review and meta-analysis by the European lung Cancer working Party for the International Association for the study of lung Cancer staging project. J Thorac Oncol.

[12] Im HJ, Pak K, Cheon GJ, Kang KW, Kim SJ, Kim IJ (2015). Prognostic value of volumetric parameters of (18) F-FDG PET in non-small cell lung cancer: a meta-analysis. Eur J Nucl Med Mol Imaging.

[13] Lim R, Eaton A, Lee NY, Setton J, Ohri N, Rao S (2012). 18F-FDG PET/CT metabolic tumor volume and total lesion glycolysis predict outcome in oropharyngeal squamous cell carcinoma. J Nucl Med.

[14] Chung HH, Kim JW, Han KH, Eo JS, Kang KW, Park NH (2011). Prognostic value of metabolic tumor volume measured by FDG-PET/CT in patients with cervical cancer. Gynecol Oncol.

[15] Hyun SH, Ahn HK, Kim H, Ahn MJ, Park K, Ahn YC (2014). Volume-based assessment by (18) F-FDG PET/CT predicts survival in patients with stage III non-small-cell lung cancer. Eur J Nucl Med Mol Imaging.

[16] Lemarignier C, Di Fiore F, Marre C, Hapdey S, Modzelewski R, Gouel P (2014). Pretreatment metabolic tumour volume is predictive of disease-free survival and overall survival in patients with oesophageal squamous cell carcinoma. Eur J Nucl Med Mol Imaging.

[17] Tomiyoshi K, Amed K, Muhammad S, Higuchi T, Inoue T, Endo K (1997). Synthesis of new fluorine-18 labeled amino acid radiopharmaceutical: L-F-α-methyl tyrosine using separation and purification system. Nucl Med Commun.

[18] Wiriyasermkul P, Nagamori S, Tominaga H, Oriuchi N, Kaira K, Nakao H (2012). Transport of 3-fluoro-L-α-methyl-tyrosine by tumor-upregulated L-type amino acid transporter 1: a cause of the tumor uptake in PET. J Nucl Med.

[19] Kaira K, Oriuchi N, Otani Y, Shimizu K, Tanaka S, Imai H (2007). Fluorine-18-α-methyltyrosine positron emission tomography for diagnosis and staging of lung cancer: a clinicopathological study. Clin Cancer Res.

[20] Wei L, Tominaga H, Ohgaki R, Wiriyasermkul P, Hagiwara K, Okuda S (2016). Specific transport of 3-fluoro-l-α-methyl-tyrosine by LAT1 explains its specificity to malignant tumors in imaging. Cancer Sci.

[21] Kim M, Achmad A, Higuchi T, Arisaka Y, Yokoo H, Yokoo S (2015). Effects of intratumoral inflammatory process on 18F-FDG uptake: pathologic and comparative study with 18F-fluoro-α-methyltyrosine PET/CT in oral squamous cell carcinoma. J Nucl Med.

[22] Morita M, Higuchi T, Achmad A, Tokue A, Arisaka Y, Tsushima Y (2013). Complementary roles of tumour specific PET tracer 18F-FAMT to 18F-FDG PET/CT for the assessment of bone metastasis. Eur J Nucl Med Mol Imaging..

[23] Kaira K, Oriuchi N, Shimizu K, Tominaga H, Yanagitani N, Sunaga N (2009). 18F-FMT uptake seen within primary cancer on PET helps predict outcome of non-small cell lung cancer. J Nucl Med.

[24] Kaira K, Oriuchi N, Yanagitani N, Sunaga N, Ishizuka T, Mori M (2010). Assessment of therapy response in lung cancer with ^18^F-α-methyl tyrosine PET. AJR Am J Roentgenol.

[25] Kaira K, Oriuchi N, Shimizu K, Imai H, Tominaga H, Yanagitani N (2010). Comparison of L-type amino acid transporter 1 expression and L-[3-18F]-α-methyl tyrosine uptake in outcome of non-small cell lung cancer. Nucl Med Biol.

[26] Davison J, Mercier G, Russo G, Subramaniam RM (2013). PET-based primary tumor volumetric parameters and survival of patients with non-small cell lung carcinoma. AJR Am J Roentgenol.

[27] Chen HH, Chiu NT, Su WC, Guo HR, Lee BF (2012). Prognostic value of whole-body total lesion glycolysis at pretreatment FDG PET/CT in non-small cell lung cancer. Radiology.

[28] Liao S, Penney BC, Wroblewski K, Zhang H, Simon CA, Kampalath R (2012). Prognostic value of metabolic tumor burden on 18F-FDG PET in nonsurgical patients with non-small cell lung cancer. Eur J Nucl Med Mol Imaging.

[29] Chung HW, Lee KY, Kim HJ, Kim WS, So Y (2014). FDG PET/CT metabolic tumor volume and total lesion glycolysis predict prognosis in patients with advanced lung adenocarcinoma. J Cancer Res Clin Oncol.

[30] Bazan JG, Duan F, Snyder BS, Horng D, Graves EE, Siegel BA (2017). Metabolic tumor volume predicts overall survival and local control in patients with stage III non-small cell lung cancer treated in ACRIN 6668/RTOG 0235. Eur J Nucl Med Mol Imaging.

[31] Carlier T, Bailly C (2015). State-Of-The-Art and Recent Advances in Quantification for Therapeutic Follow-Up in Oncology Using PET. Front Med (Lausanne)..

[32] Moon SH, Hyun SH, Choi JY (2013). Prognostic significance of volume-based PET parameters in cancer patients. Korean J Radiol.

[33] van Baardwijk A, Bosmans G, Boersma L, Buijsen J, Wanders S, Hochstenbag M (2007). PET-CT-based auto-contouring in non-small-cell lung cancer correlates with pathology and reduces interobserver variability in the delineation of the primary tumor and involved nodal volumes. Int J Radiat Oncol Biol Phys..

[34] Kim M, Higuchi T, Arisaka Y, Achmad A, Tokue A, Tominaga H (2013). Clinical significance of ^18^F-α-methyl tyrosine PET/CT for the detection of bone marrow invasion in patients with oral squamous cell carcinoma: comparison with ^18^F-FDG PET/CT and MRI. Ann Nucl Med.

[35] Suzuki S, Kaira K, Ohshima Y, Ishioka NS, Sohda M, Yokobori T (2014). Biological significance of fluorine-18-α-methyltyrosine (FAMT) uptake on PET in patients with oesophageal cancer. Br J Cancer.

